# Evaluating the efficacy of a telehealth management model for chronic diabetes in resource-constrained regions

**DOI:** 10.3389/fendo.2026.1812377

**Published:** 2026-04-23

**Authors:** Min Chen, Jing Fu, Puxian Tang, Dizhi Liu, Ying Wang, Xia Huang, Yan Wen, Chenghua Liu, Kai Zhou, Ge Yu, Yan Zhou

**Affiliations:** 1 Department of Nephrology and Rheumatology/Immunology, People’s Hospital of Dafang, Bijie, Guizhou, China; 2 General Practice Department, Beijing Hospital, National Center of Gerontology; National Clinical Research Center for Gerontology; The Key Laboratory of Geriatrics of NHC; Institute of Geriatric Medicine, Chinese Academy of Medical Sciences, Beijing, China; 3 Medical Department, Beijing Hospital, National Center of Gerontology; National Clinical Research Center for Gerontology; The Key Laboratory of Geriatrics of NHC; Institute of Geriatric Medicine, Chinese Academy of Medical Sciences, Beijing, China; 4 The Key Laboratory of Geriatrics, Beijing Institute of Geriatrics, Institute of Geriatric Medicine, Chinese Academy of Medical Sciences, Beijing Hospital/National Center of Gerontology of National Health Commission, Beijing, China; 5 Department of Endocrinology, People’s Hospital of Dafang, Bijie, Guizhou, China; 6 Department of Geriatrics and Clinical Nutrition, People’s Hospital of Dafang, Bijie, Guizhou, China; 7 The Health Center of Huannitang Town, Bijie, Guizhou, China; 8 The Health Center of Daxi Town, Bijie, Guizhou, China; 9 The Health Center of Xiaotun Town, Bijie, Guizhou, China

**Keywords:** chronic disease management, diabetes mellitus, glycemic control, integrated care, telehealth

## Abstract

**Objective:**

To assess the effects of a telehealth-enhanced integrated county-township-village management model (hereafter referred to as the telehealth management model) on metabolic indicators and chronic complications of rural-dwelling individuals with diabetes mellitus (DM), seeking an effective chronic disease management approach for regions with limited medical resources.

**Methods:**

An exploratory quasi-experimental study was conducted in Dafang County, Guizhou Province. Three townships were assigned to the management group, while the remaining townships constituted the control group. The management group participated in a 12-month diabetes intervention using a telemedicine platform for comprehensive care, while the control group received standard outpatient follow-up. Key indicators measured before and after the intervention included fasting blood glucose (FBG), 2-hour postprandial blood glucose (2hPG), haemoglobin A1c (HbA1c), blood lipids, blood pressure, body mass index (BMI), the incidence of new chronic complications, and the pass rate on a diabetes knowledge assessment. Statistical analyses, including descriptive analyses for patient characteristics, multivariable regression for associations and adjusted effects, subgroup and restricted cubic spline analyses for heterogeneous and nonlinear relationships, and a propensity score matching combined with difference-in-differences (PSM-DID) approach to estimate the causal effect of the intervention, were performed.

**Results:**

In this study including 215 patients (88 in the management group and 127 in the control group), compared with the control group, the management group showed significant improvements after 12 months, including lower FBG (8.97 vs. 10.77), 2hPG (13.30 vs. 16.96), HbA1c (7.84 vs. 9.45), triglyceride (TG, 1.84 vs. 2.43), and BMI (23.94 vs. 26.09) levels and higher high-density lipoprotein cholesterol (HDL-C, 1.26 vs. 1.10) levels (all *P* < 0.05). They also had fewer new chronic complications (8.0% vs. 29.9% in the control group; *P* < 0.001). Multivariate analysis and PSM-DID analysis demonstrated an association between the management group and improved glycaemic control, and a reduction in the incidence of new complications.

**Conclusion:**

Compared to standard of care, the telehealth management model improved patients’ metabolic indicators, decreased complication risks, and strengthened primary health care services. This model provides a replicable example for the scalable implementation of integrated diabetes management in similar underdeveloped regions.

## Introduction

Diabetes is recognized as a significant global health challenge, affecting 11.11% (589 million) of adults worldwide in 2024, and is predicted to increase to 12.96% (853 million) by 2050. Diabetes currently accounts for 11.9% of global health expenditures (1). China has around 150 million people living with diabetes—approximately 25.5% of the global total— making it the largest diabetic population worldwide, mostly affected by type 2 diabetes. Between 2013–2018, the prevalence of diabetes among Chinese adults increased from 10.9% to 12.4%, diabetes awareness in China slightly increased from 36.5% to 36.7%, treatment rates rose from 32.2% to 32.9%, and control rates improved from 49.2% to 50.1% (2, 3). Despite these gains, the control rates remain low, especially in rural areas compared with urban areas (4, 5). Diabetes places a significant economic burden on China’s health care system, with per capita costs projected to increase from 231 to 414 USD between 2020 and 2030 (6). In response to this considerable public health challenge, the State Council has identified diabetes as a focal point within the “Healthy China 2030” initiative (7).

Significant disparities exist among various regions in China concerning health literacy, the quality and accessibility of diabetes management, and resource allocation. Despite the high prevalence of diabetes, public awareness of its risk factors and the importance of preventive measures remain insufficient, particularly in rural and underdeveloped areas. According to the literature, in rural areas of southwestern China in 2022, the prevalence of diabetes among individuals aged 35 and above was 7.6%, with awareness, treatment, and control rates of 57.3%, 48.4%, and 21.0%, respectively (8). Although the prevalence of diabetes in rural areas is relatively lower than that in urban areas, the mortality rate is higher (9), suggesting that diabetes management in rural regions continues to face significant challenges.

In recent years, various regions in China have been actively investigating effective models for diabetes management by integrating medical resources and enhancing the processes of screening, diagnosis, treatment, and follow-up care for patients with diabetes. Presently, the predominant diabetes management models in China include the hospital-based management model, the community-based management model, and the “hospital–community integrated” management model (10). The hospital-based model is advantageous because of the presence of medical personnel with a high level of expertise in diabetes diagnosis and treatment. However, it is limited by the challenges faced by physicians in providing comprehensive patient education and long-term follow-up care, owing to their substantial workloads (11). Conversely, the community-based model is characterized by high adherence to follow-up and low rates of patient attrition, yet it is hindered by the lower professional competency of community health care workers and diminished patient trust (12). The “hospital–community integrated” management model seeks to amalgamate the strengths of the two preceding models, primarily through bidirectional referral and county-township-village coordination (13, 14).

The implementation of this integrated model faces challenges, including a lack of standardization across different diabetes management systems and limited research in rural areas, as most related studies focus on urban settings (15). In resource-limited rural areas of western China, there is an urgent need for a diabetes management model suited to local conditions.

This study aims to address the limited evidence concerning effective management models in resource-constrained regions by developing and evaluating a telehealth management model for diabetes care in Dafang County, a resource-limited rural area in Guizhou Province, located in western China. The model employs a telemedicine platform to establish an integrated county-township-village framework. Its effectiveness was assessed through within-group and between-group comparisons, evaluating a range of outcomes, including metabolic indicators (e.g., glycaemic control), diabetes-related complications, and health care provider competency. This study provides a replicable example for the scalable implementation of integrated diabetes management in similar underdeveloped regions.

## Methods

### Study design

This study is an exploratory quasi-experimental study using a nonblinded, nonrandomized, two-arm comparative design. On the basis of a comprehensive assessment of township-level conditions—including factors such as expressed willingness and readiness to implement the new telehealth management model, patient volume, the staffing capacity of local community health care centres and existing health care infrastructure—three townships (Daxi Town, Xiaotun Township, and Huangnitang Town) in Dafang County, Guizhou Province, were selected to serve as management groups for the implementation of the telehealth management model for diabetes patients. The remaining townships were designated as the control group. Patients previously diagnosed with diabetes who visited the local community health care centre between January 2023 and January 2024 and met the specified inclusion and exclusion criteria were included in this study.

Owing to the quasi-experimental design of the study, randomization between the management and control groups was not feasible. As a result, baseline comparability of outcome variables could not be guaranteed, rendering the analysis vulnerable to confounding and selection bias. Consequently, a pre–post comparison within the management group or a cross-sectional comparison between the two groups would be inadequate for producing an unbiased estimate of the management effect. To address these methodological limitations, we employed a propensity score matching combined with difference-in-differences (PSM-DID) approach. This analytical strategy was implemented to statistically adjust for baseline differences and to mitigate the impact of confounding variables, thereby enhancing the causal inferences regarding the effect of the telehealth management model. A comprehensive description of the PSM-DID methodology is provided in the Statistical Analysis section.

This study was carried out in compliance with the Declaration of Helsinki and was approved by the Ethics Committee of Dafang County People’s Hospital, Guizhou Province (Approval No. 20231001), and informed consent was obtained from all participants.

### Participants

Participants were recruited from the outpatient clinics of the local township health centre. The inclusion criteria were as follows: (1) a diagnosis of type 2 diabetes mellitus in accordance with the American Diabetes Association (ADA) criteria; (2) a disease duration exceeding one year; (3) an age range of 18 to 70 years, with a status as a permanent rural resident, defined as having resided in rural areas for a cumulative period exceeding six months at the time of enrolment; (4) clear consciousness and the ability to communicate effectively; and (5) provision of signed informed consent. The exclusion criteria were as follows: (1) the presence of severe heart, liver, or kidney diseases; (2) a diagnosis of gestational diabetes mellitus; (3) relocation away from their permanent residence following enrolment; and (4) lost to follow-up.

**Figure 1:**
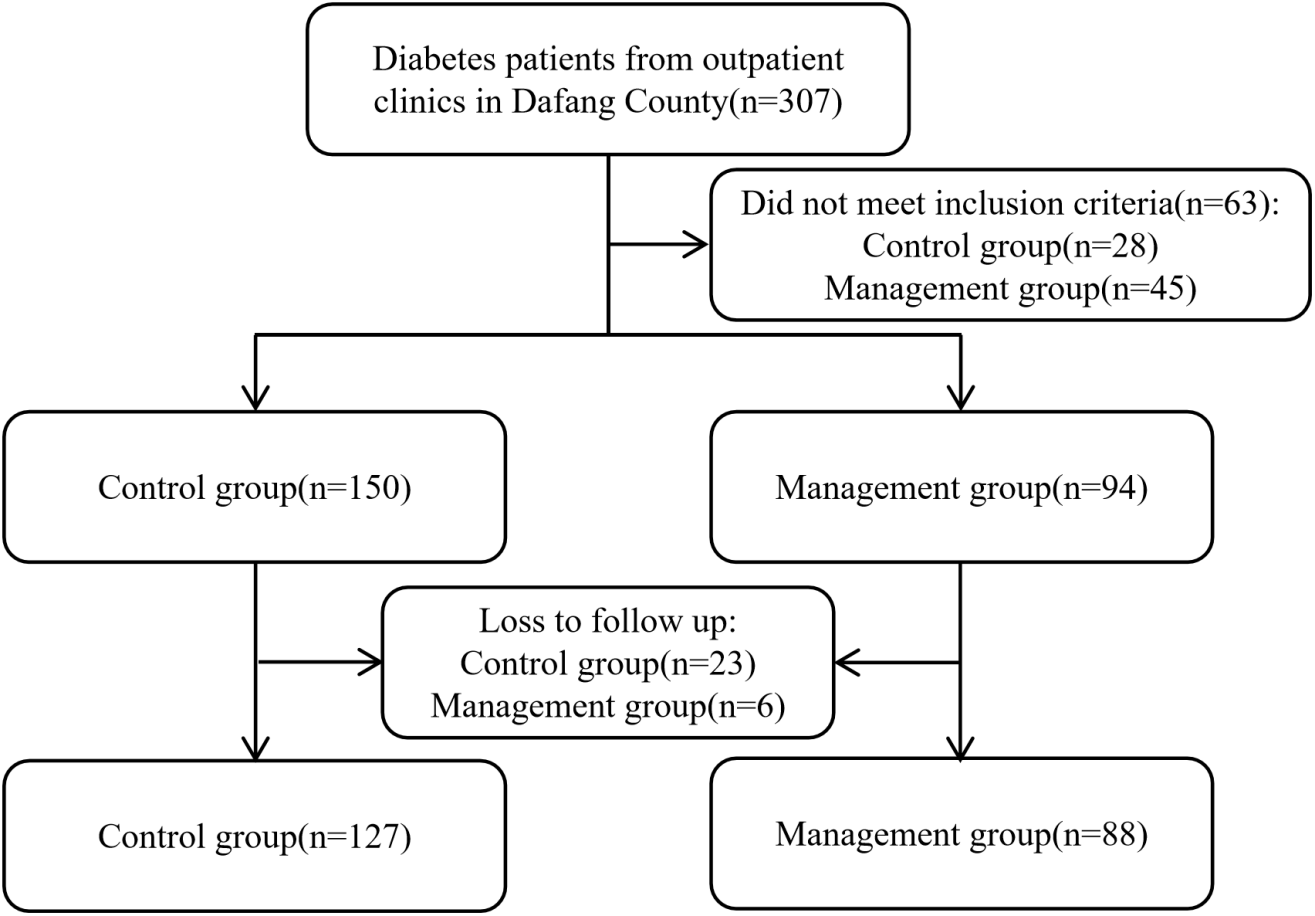
Flowchart of participant enrolment.

Initially, 94 participants were enrolled in the management group and 150 in the control group, with all participants being followed for a duration of 12 months. By the conclusion of the follow-up period, 6 participants were lost from the management group, resulting in a loss rate of 6.4%, while 23 participants were lost from the control group, corresponding to a loss rate of 15.3%. Therefore, the final analysis included 88 participants in the management group and 127 in the control group, and the flowchart of participant enrollment is presented in **Figure 1**.

### Formation of the management team

A telehealth-enhanced integrated management model for diabetes was developed, centred on a “county-township-village” framework. This model was anchored by the people’s hospital of Dafang Country and involved collaboration with medical institutions at the county, township, and village levels. The specific components of this model included the following: (1) The establishment of a comprehensive management and intervention team spanning the county, township, and village levels. The team comprised dedicated personnel from the county-level research group alongside medical staff from the township and village levels. (2) The establishment of an online collaborative working mechanism that utilized a telemedicine service management platform. This platform facilitated online teaching and guidance by county-level hospital experts, thereby directly enhancing the professional capabilities of rural medical personnel. Additionally, the platform enabled online collaborative diagnosis and treatment among medical staff at the county, township, and village levels, allowing them to jointly formulate and optimize diabetes management plans for rural patients. (3) The conduction of standardized training and assessment of all team members prior to commencement of the study. The training content included guidance on diabetes medication, instructions on insulin injection, health education, and practical case management. Periodic feedback sessions and supplementary training were also provided. At the conclusion of the follow-up period, a comprehensive assessment was conducted to evaluate the team members’ mastery of the relevant knowledge.

Further details of this telehealth management model are provided in the **Supplementary Data**.

### Patient management strategies

The treatment regimen for all enrolled participants was developed in accordance with the Chinese Guidelines for the Prevention and Treatment of Type 2 Diabetes (2020 edition) and the National Guidelines for Primary Diabetes Prevention and Management (2022 edition). The control group received standard outpatient follow-up care, wherein patients independently attended local clinics and were provided with health education.

The management group was administered care through the “integrated patient self-management education and support model for diabetes”, which encompassed a structured process involving assessment, goal setting, implementation, and feedback. (1) Assessment: Upon enrolment, each participant participated in an individual interview with a team member to collect foundational information and evaluate their lifestyle, diabetes self-management knowledge, and adherence levels. (2) Goal Setting: Based on the assessment outcomes, short-term objectives (enhancing knowledge and self-management skills) and long-term objectives (improving clinical outcomes) were established for each participant. (3) Implementation and Feedback: Participants were provided with personalized recommendations regarding medication and lifestyle, alongside relevant technical and psychological support, tailored to their specific circumstances. Regular follow-ups were systematically implemented, comprising monthly routine check-ups, quarterly screenings for health risk factors, and periodic health education sessions.

### Indicator measurement and calculation

The medical team conducted standardized individual interviews of the participants to gather data on their demographics and medical histories, including age, sex, ethnicity, educational attainment, smoking history, previous medical conditions, and family medical history. Verification of name, age, and ethnicity was performed using identity cards or household registration documents. The chronic complications associated with diabetes considered in this study are diabetic kidney disease, diabetic retinopathy, diabetic neuropathy, diabetic lower extremity arterial disease, and diabetic foot. Additionally, data on acute complications of diabetes, namely diabetic ketoacidosis and a hyperosmolar hyperglycaemic state, were collected. Adherence to medical advice was assessed on the basis of attendance at scheduled follow-up visits and categorized as follows: “good” (≤1 missed visit), “fair” (2–4 missed visits), or “poor” (≥5 missed visits).

The medical team conducted anthropometric and physiological assessments by measuring participants’ height, weight, systolic blood pressure (SBP), and diastolic blood pressure (DBP) in accordance with established protocols. In the morning, fasting peripheral venous blood samples were obtained from the participants to analyse their FBG, HbA1c, TG, total cholesterol (TC), low-density lipoprotein cholesterol (LDL-C), and HDL-C levels. 2hPG levels were determined following the oral administration of 75 g of an anhydrous glucose solution. BMI was computed as the weight in kilograms divided by the square of the height in metres (kg/m²). The homeostatic model assessment of insulin resistance (HOMA-IR) was calculated using the formula [FBG (mmol/L) × fasting insulin (μU/mL)]/22.5. Similarly, the homeostatic model assessment of β-cell function (HOMA-β) was derived using the formula: [20 × fasting insulin (μU/mL)]/[FBG (mmol/L) – 3.5].

### Statistical analysis

#### Data preprocessing and descriptive analysis

Data processing was conducted utilizing SPSS version 25.0 and R software version 4.5.2. For variables exhibiting a limited number of outliers, whether missing or falling outside the predefined range, the mean imputation method was applied to continuous variables, while the median was employed for categorical variables. Measurement data following a Gaussian distribution are expressed as the mean ± standard deviation ( $\bar{\text{x}}$ ± S), with intergroup comparisons conducted via the t test. Categorical data are presented as frequencies (percentages), with intergroup comparisons executed using the χ² test or Fisher’s exact test.

#### Multivariable regression analysis

To evaluate the differences between pre- and postmanagement, multiple linear regression and logistic regression analyses were employed. The findings are presented as regression coefficients (b values), odds ratios (ORs), and 95% confidence intervals (CIs). The assignment of variables for multivariable analysis is detailed in **Table 1**. To assess potential multicollinearity among the variables, the variance inflation factor (VIF) was calculated, with a VIF value of ≥ 5 indicating significant multicollinearity.

**Table 1:** Multivariable analysis variable assignment.

Variable	Assignment
Group	1=Control group, 2=Management group
Time	0=Baseline, 1=Endpoint
Sex	0=Female, 1=Male
Ethnicity	0=Han, 1=Ethnic minority
Smoker	0=No, 1=Yes
Baseline chronic complication	0=No, 1=Yes
Adherence to medical advice	1=Good, 2=Fair, 3=Poor
New-onset chronic complication	0=No, 1=Yes

#### Subgroup analysis and nonlinear relationship exploration

Subgroup analyses were conducted utilizing the forestploter package to investigate the association between diabetes management and endpoint indicators across diverse subgroups, including age (<45, 45–65, >65 years), sex (male, female), smoking status (yes, no), baseline complications (yes, no), and groups categorized by the median (lower/higher group) for baseline fasting blood glucose, 2-hour postprandial glucose, and HbA1c levels. Additionally, restricted cubic spline (RCS) modelling and analysis were performed using the ggrcs package to examine potential nonlinear relationships between diabetes management and various indicators. A *P* < 0.05 (two-tailed) was considered to indicate statistical significance.

#### PSM-DID

PSM aims to reduce confounding bias from observable covariates by pairing subjects with similar propensity scores between the management and control groups. The fundamental principle of DID is to utilize longitudinal data from comparable management and control groups before and after implementation of the management, whereby the management effect is estimated by comparing the difference in average changes over time between the two groups.

To address potential endogeneity (wherein patients with poorer baseline health were more likely to participate in the management program), we combined PSM and DID in a sequential analytical framework. PSM matches subjects by premanagement traits to address selection bias from observed confounders. However, it does not account for unobserved time-invariant factors. DID complements this by leveraging pre- and postintervention data to cancel out time-invariant unobservables under the parallel trends assumption. This combined approach improves comparability and controls for constant unobserved confounders, strengthening the study’s internal validity and yielding more robust estimates of the management effect on glycaemic outcomes.

Specifically, we used three different PSM modalities (1:4 nearest neighbour matching; optimal pair matching, and optimal full matching) to estimate the average treatment effect on the treated (ATT). Propensity score distributions before and after matching were visualized using histograms and density plots. Subsequently, DID analysis was conducted on the matched samples generated by each PSM modality to derive the final estimates.

## Results

### Baseline characteristics of participants and pre- and postmanagement changes

This study included a total of 215 participants, who were divided into 88 individuals in the management group and 127 individuals in the control group. Upon analysis, no statistically significant differences were observed between the two groups concerning baseline demographic variables, including age, sex, and ethnicity, or health indicators such as glucose metabolism, blood lipid levels, and blood pressure. There was a significant difference in chronic complications between the control and management groups (65.9% vs. 46.5%, *P* = 0.005).

After a 12-month management period, a univariate analysis was conducted to compare pre- and postintervention data for the control and management groups. The control group, with standard outpatient follow-up, showed a significant decrease in fasting blood glucose (10.77 vs. 12.08, *P* = 0.037), while the other indicators were mostly unchanged. The management group, which received an additional 12 months of management, experienced significant reductions in FBG (8.97 vs. 12.21), 2hBG (13.30 vs. 17.75), HbA1c (7.84 vs. 10.01), fasting insulin (69.11 vs. 85.76), HOMA-IR (26.8 vs. 46.8), TG (1.84 vs. 3.14), LDL-C (2.81 vs. 3.08), BMI (23.94 vs. 25.23), SBP (124.01 vs. 133.58), and DBP (76.76 vs. 81.98), along with an increase in HDL-C (1.26 vs. 1.07). They also had a lower incidence of new chronic complications than the control group did (8.0% vs. 29.9%). The detailed data and statistical results are shown in **Tables 2** and **3**.

**Table 2:** Summary of Pre- and postmanagement information of the study.

Variable	Control group		Change from baseline	Management group		Change from baseline
	Baseline	Endpoint		Baseline	Endpoint	
Sex						
Female	67(52.8)			47(53.4)		
Male	60(47.2)			41(46.6)		
Age	51.16 ± 9.5			51.24 ± 9.6		
Ethnicity						
Han ethinicity	105(82.7)			73(83.0)		
Ethnic minority	22(17.3)			15(17.0)		
Educational attainment						
Primary school and below	91(71.7)			49(55.7)		
Junior middle school	22(17.3)			19(21.6)		
Senior middle school and above	14(11.0)			20(22.7)		
Smoker						
No	78(61.4)			61(69.3)		
Yes	49(38.6)			27(30.7)		
Baseline chronic complication						
No	68(53.5)			30(34.1)		
Yes	59(46.5)			58(65.9)		
New-onset chronic complication						
No		89(70.1)			81(92.0)	
Yes		38(29.9)			7(8.0)	
New-onset acute complication						
No		111(87.4)			77(87.5)	
Yes		13(10.2)			5(5.7)	
Adherence to medical advice						
Good		61(48.0)		54(61.4)		
Fair		44(34.6)		21(23.9)		
Poor		22(17.3)		13(14.8)		
FBG (mmol/L)	12.08 ± 5.37	10.77 ± 4.55	-1.31 ± 4.85	12.21 ± 5.06	8.97 ± 3.69	-3.23 ± 5.53
2hPG (mmol/l)	16.70 ± 6.71	16.96 ± 6.84	0.26 ± 6.06	17.75 ± 5.58	13.30 ± 5.28	-4.45 ± 6.86
HbA1c (%)	9.43 ± 2.65	9.45 ± 2.52	0.03 ± 2.56	10.01 ± 2.50	7.84 ± 2.04	-2.17 ± 3.11
Fasting insulin (µIU/ml)	80.14 ± 58.11	78.13 ± 52.18	-2.01 ± 64.95	85.76 ± 59.73	69.11 ± 35.11	-16.65 ± 60.00
C-peptide (ng/ml)	0.96 ± 1.78	0.88 ± 1.23	-0.08 ± 1.18	0.61 ± 0.47	0.68 ± 0.33	0.06 ± 0.41
HOMA-β	282.2 ± 278.3	411.8 ± 828.6	132.0 ± 852.4	374.7 ± 826.5	384.5 ± 360.8	9.8 ± 857.3
HOMA-IR	40.4 ± 30.9	36.6 ± 26.9	-3.8 ± 34.9	46.8 ± 45.6	26.8 ± 15.2	-20.1 ± 44.4
TG (mmol/L)	3.19 ± 4.11	2.43 ± 5.44	0.25 ± 6.30	3.14 ± 5.38	1.84 ± 1.10	-1.30 ± 5.20
TC (mmol/L)	4.73 ± 1.76	4.66 ± 2.09	-0.06 ± 2.67	4.79 ± 2.24	4.23 ± 1.46	-0.56 ± 2.16
LDL-C (mmol/L)	3.08 ± 4.73	3.17 ± 4.97	0.09 ± 1.19	3.08 ± 0.98	2.81 ± 1.11	-0.27 ± 1.10
HDL-C (mmol/L)	1.07 ± 0.37	1.10 ± 0.50	0.03 ± 0.43	1.07 ± 0.29	1.26 ± 0.49	0.19 ± 0.51
BMI (kg/m^2^)	25.00 ± 4.41	26.09 ± 4.48	1.09 ± 2.81	25.23 ± 3.69	23.94 ± 3.46	-1.29 ± 2.05
SBP (mmHg)	130.83 ± 18.16	134.93 ± 19.08	4.10 ± 20.91	133.58 ± 18.71	124.01 ± 18.20	-9.57 ± 21.79
DBP (mmHg)	81.32 ± 11.08	83.34 ± 10.34	2.02 ± 12.75	81.98 ± 11.28	76.76 ± 8.48	-5.22 ± 11.44

The data are presented as n (%) or mean ± SD, n=215.

**Table 3:** Statistical outcomes of univariate analysis.

Variable	*P* Value			
	Baseline differences	Change from baseline		Endpoint differences
		Control	Management	
Sex	0.925			
Age	0.951			
Ethnicity	0.958			
Educational attainment	0.022^a^			
Smoker	0.233			
Baseline chronic complication	0.005			
New-onset chronic complication				<0.001
New-onset acute complication^a^				0.146
Adherence to medical advice				0.140
FBG (mmol/L)	0.861	0.037	<0.001	0.002
2hPG (mmol/L)	0.227	0.759	<0.001	<0.001
HbA1c (%)	0.107	0.939	<0.001	<0.001
Fasting insulin (µIU/ml)	0.491	0.772	0.025	0.159
C-peptide (ng/ml)	0.074	0.668	0.297	0.134
HOMA-β	0.245	0.097	0.919	0.772
HOMA-IR	0.221	0.294	<0.001	0.002
TG (mmol/L)	0.947	0.686	0.028	0.007
TC (mmol/L)	0.815	0.793	0.051	0.095
LDL-C (mmol/L)	0.998	0.877	0.090	0.500
HDL-C (mmol/L)	0.925	0.557	0.002	0.023
BMI (kg/m^2^)	0.688	0.052	0.018	<0.001
SBP (mmHg)	0.282	0.080	0.001	<0.001
DBP (mmHg)	0.669	0.134	0.001	<0.001

^a^ indicates the Fisher–Freeman–Halton exact test.

### Multivariable analysis of changes from baseline to follow-up

A multivariable analysis was conducted to assess the independent effect of diabetes management on glucose metabolism indicators, considering factors such as sex, age, ethnicity, smoking status, education status, chronic complications, adherence to medical advice, and baseline glycaemic and lipid levels. After adjustment for confounders, participants in the diabetes management group significantly improved compared with those in the control group, with reductions in endpoint fasting blood glucose (β = -1.91; 95% CI: -2.98, -0.84), endpoint 2-hour postprandial blood glucose (β = -3.41; 95% CI: -4.90, -1.93), endpoint HbA1c (β = -1.55; 95% CI: -2.08, -1.01), and the incidence of new-onset chronic complications (OR = 0.246; 95% CI: 0.092, 0.656) (**Table 4**).

**Table 4:** Statistical outcomes of multivariate analysis.

Dependent variable	Independent variable	β/OR value (95% CI)	*P* value
FBG postintervention	Diabetes management	-1.91(-2.98, -0.84)	0.001
	FBG preintervention	0.27(0.15, 0.39)	<0.001
	Adherence to medical advice	1.35(0.67, 2.03)	<0.001
2hPG postintervention	Diabetes management	-3.41(-4.90, -1.93)	<0.001
	FBG preintervention	0.23(0.07, 0.39)	0.006
	2hPG preintervention	0.38(0.23, 0.54)	<0.001
	HbA1c preintervention	-0.45(-0.82, -0.09)	0.015
	Adherence to medical advice	2.15(1.21, 3.10)	<0.001
HbA1c postintervention	Diabetes management	-1.55(-2.08, -1.01)	<0.001
	FBG preintervention	0.13(0.07, 0.19)	<0.001
	2hPG preintervention	0.06(0.00, 0.11)	0.04
	Adherence to medical advice	1.03(0.69, 1.38)	<0.001
New-onset chronic complication^a^	Diabetes management	0.25(0.092, 0.69)	0.008
	SBP preintervention	1.04(1.00, 1.08)	0.029
	Baseline chronic complication	0.15(0.05, 0.42)	<0.001
	Adherence to medical advice	2.27(1.33, 3.87)	0.003

^a^ the effect estimate for this variable derived from multivariable regression is expressed as an odds ratio (OR).

The multivariable analysis revealed a significant link between participants’ initial glucose metabolism levels, their adherence to medical advice, and changes in their endpoint glucose metabolism indicators. To test the robustness of these findings, the following analyses were conducted: (1) Subgroup analysis to evaluate the effect of diabetes management on the endpoint of glucose metabolism across different subgroups; and (2) Restricted cubic spline models to visualize exposure–response curves for variables with significant interactions found in the subgroup analysis.

**Figure 2:**
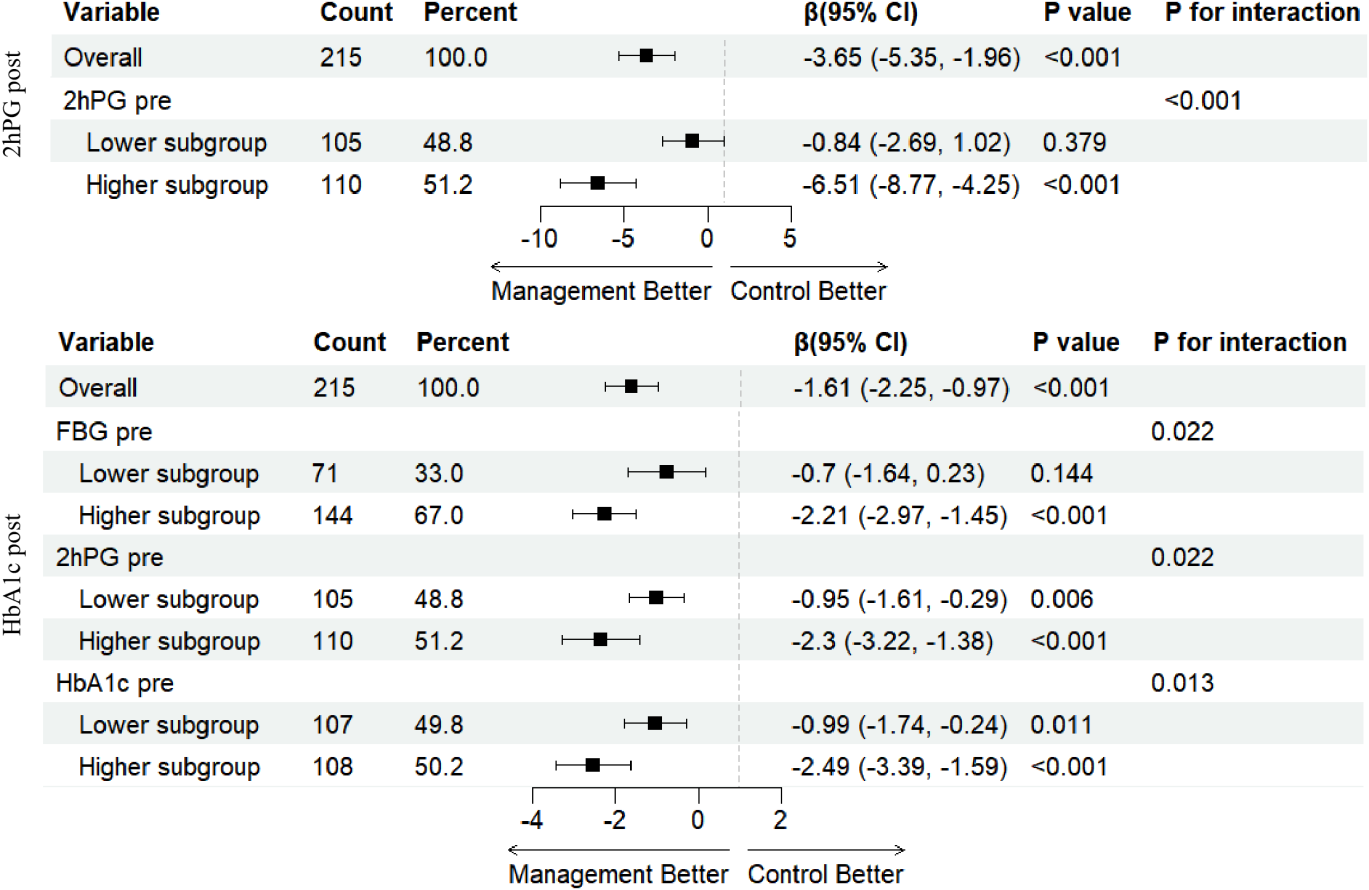
Greater effectiveness of diabetes management in patients with worse baseline glycaemic control. Adjusted for sex, smoking history, age, baseline fasting blood glucose, baseline 2-hour postprandial blood glucose, baseline HbA1c, baseline chronic complications, and adherence to medical advice. Data are presented as regression coefficients (β) with 95% confidence intervals (CIs).

The subgroup analysis revealed differences in diabetes management effectiveness based on age and baseline glucose metabolism. Participants with high baseline 2-hour postprandial blood glucose levels showed significant decreases, β (95% CI) = -6.51 (-8.77, -4.25), *P* < 0.001, whereas those with lower baseline levels did not experience significant changes. Similarly, those with high baseline FBG (β (95% CI) = -2.21 (-2.97, -1.45), *P* < 0.001), 2hPG (β (95% CI) = -2.3 (-3.22, -1.38), *P* < 0.001), and HbA1c (β (95% CI) = -2.49 (-3.39, -1.59), *P* < 0.001) experienced significant reductions in endpoint HbA1c levels (**Figure 2**).

**Figure 3:**
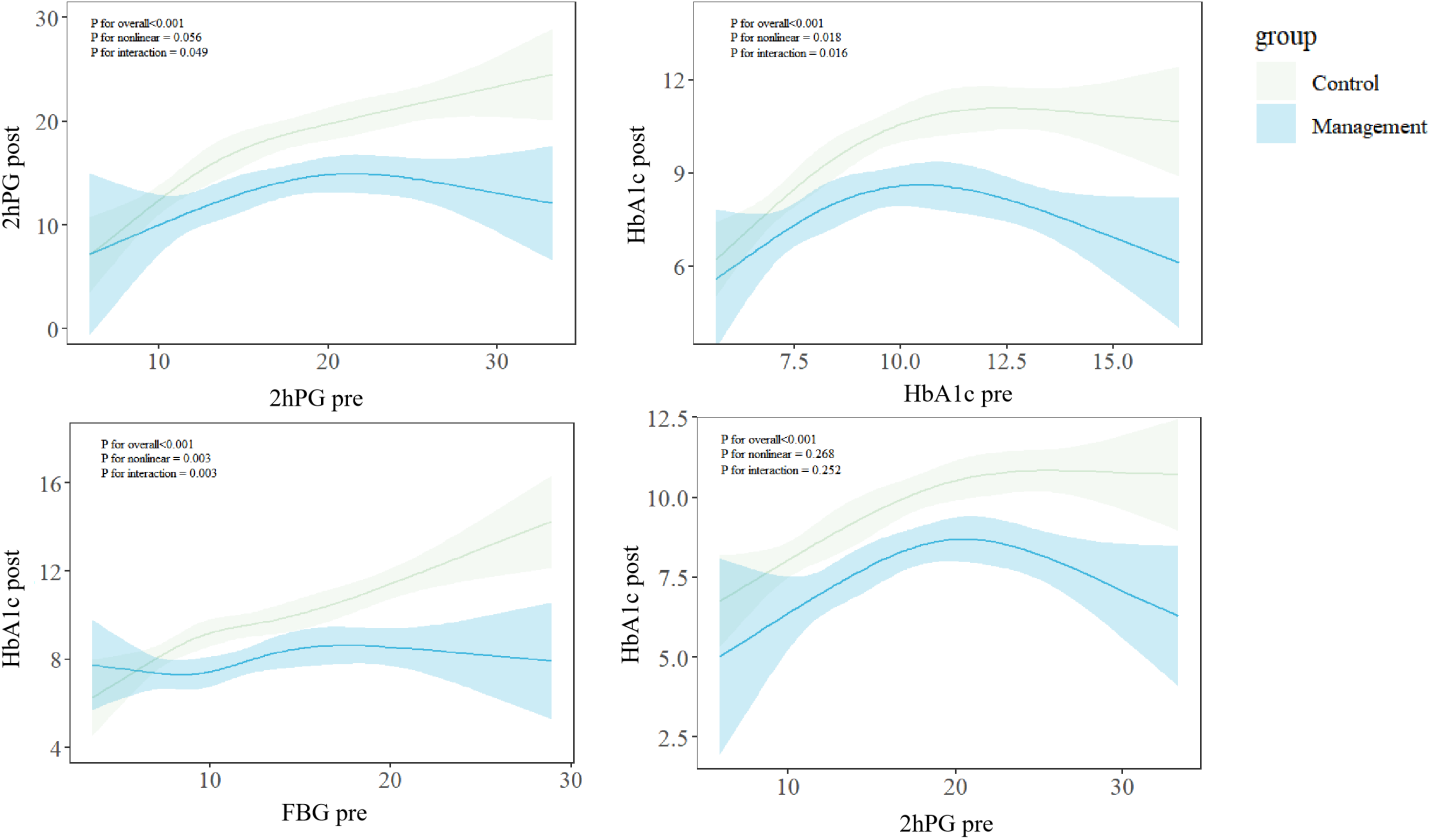
Exposure–response curves showing greater glycaemic reduction with management at higher baseline levels. Fitting and comparison of restricted cubic spline models for control and management groups. *P* for overall: *P* value for the overall association between independent and dependent variables; *P* for nonlinear: *P* value for the nonlinear trend; *P* for interaction: *P* value for the interaction between two groups.

The restricted cubic spline model revealed that participants with higher initial glucose metabolism indicators had greater decreases in their endpoint indicators after diabetes management than those in the control group did. Significant interactions were found between baseline and endpoint 2hPG, baseline FBG, and baseline HbA1c with endpoint HbA1c (all *P* values for interactions < 0.05) (**Figure 3**).

### PSM-DID estimates of glycaemic changes

**Figure 4:**
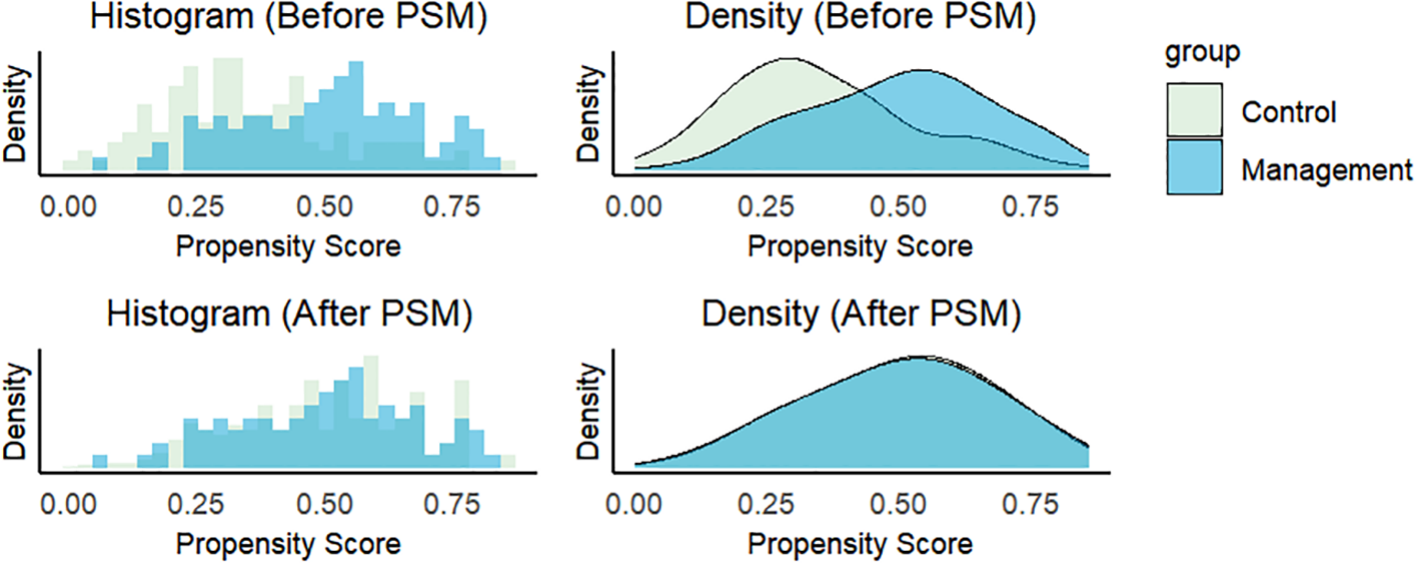
Enhanced comparability between groups after propensity score matching. Histogram and density plots of propensity scores (optimal full matching) for control and management groups.

To visualize the matching quality, propensity score distributions are shown via histograms and density plots for the control and treatment groups before and after PSM. On the basis of a comparative assessment of the three matching methods—incorporating standardized mean differences (SMDs), variance ratios (VRs), and propensity score plots—optimal full matching demonstrated the most favourable balance in both observed covariates and propensity score distributions. The propensity score plots of the optimal full matching approach are presented in **Figure 4**. The results of the SMD and VR and the propensity score plots for the other matching methodsare provided in **Supplementary Table 5** and **Supplementary Figures 3**, **S4**.

**Table 5** presents the ATT of management on glycaemic indicators among patients with diabetes, estimated using three different PSM modalities. The results of optimal full matching indicate that compared with the control group, the management group exhibited significant reductions in FBG, 2hPG and HbA1c, with corresponding ATT estimates of -2.294, -4.481 and -1.750, respectively (*P* < 0.01). The other two PSM methods also yielded significantly negative ATT estimates. Specifically, 1:4 nearest neighbour matching produced ATT values of −1.755 (FBG), −3.740 (2hPG), and −1.708 (HbA1c), while optimal pair matching yielded ATT values of −1.683 (FBG), −3.598 (2hPG), and −1.517 (HbA1c), all with *P* values < 0.01.

**Table 5:** ATT of the telehealth management model on glycaemic indicators.

Modalities		Unmatched	1:4 Nearest neighbour matching	Optimal pair matching	Optimal full matching
FBG post	Control	10.772	10.720	10.648	11.259
	Management	8.965	8.965	8.965	8.965
	ATT	-1.806	-1.755	-1.683	-2.294
	t	3.205*	-3.070**	-2.967**	-3.429**
2hBG postintervention	Control	16.969	16.981	16.839	17.721
	Management	13.240	13.240	13.240	13.240
	ATT	-3.729	-3.740	-3.598	-4.481
	t	4.514***	-4.346***	-4.020***	-4.082***
HbA1c postintervention	Control	9.458	9.556	9.365	9.598
	Management	7.848	7.848	7.848	7.848
	ATT	-1.610	-1.708	-1.517	-1.750
	t	5.160***	-5.269***	-4.595***	-4.313***

*p < 0.05, **p < 0.01, ***p < 0.001.

Optimal full matching flexibly determines the matching ratio for each treated unit by consideringthe overall distribution of propensity scores. By utilizing the entire sample, it achieves globaloptimality in covariate balance, thereby enhancing the balance between management and control groups. Therefore, we employed the matching weights derived from optimal full matching to conduct DID (**Supplementary Table 6**), and the matching weights of the other matching methods are provided in **Supplementary Tables 7** and **S8**. Prior to implementing the DID, it was necessary to conduct a parallel trends test, and theresults of the parallel trends test (**Supplementary Tables 9**-**11**) demonstrated that the interaction terms between management and covariates were essentially not significant (*P* > 0.05), suggesting that there were no systematic differences in the baseline trends of glycaemic indicators between groups and supporting the parallel trends assumption. The results of the DID are presented in **Table 6**. Following adjustment for confounding variables using PSM-DID, the implementation of the telehealth management model in rural areas was associated with a significant reduction in glycaemic indicators among patients with diabetes. Specifically, the results from optimal full matching demonstrated that, relative to the control group, the management group exhibited reductions of 2.643 mmol/L in FBG, 4.600 mmol/L in 2hPG, and 1.510% in HbA1c. Comparable significant reductions were observed with the other two PSM methods: when 1:4 nearest neighbour matching was used, the reductions were 1.873 mmol/L (FBG), 5.193 mmol/L (2hPG), and 2.921% (HbA1c), and when optimal pair matching was used, the reductions were 2.271 mmol/L (FBG), 4.598 mmol/L (2hPG), and 1.942% (HbA1c) (all *P* < 0.05).

**Table 6:** Effect of the telehealth management model on glycaemic indicators.

PSM modalities	Management vs. control		
	FBG postintervention (mmol/L)	2hBG postintervention (mmol/L)	HbA1c postintervention (%)
1:4 Nearest neighbour matching	-1.873(0.860) *	-5.193(1.099) ***	-2.921(0.440) ***
Optimal pair matching	-2.271(0.832) **	-4.598(1.120) ***	-1.942(0.444) ***
Optimal full matching	-2.643(0.832) **	-4.600(1.081) ***	-1.510(0.451) ***

*p < 0.05, **p < 0.01, ***p < 0.001; standard errors in parentheses; confounding variables are the same as those in **Table 4**.

### Enhancement in knowledge proficiency among management team members

**Figure 5:**
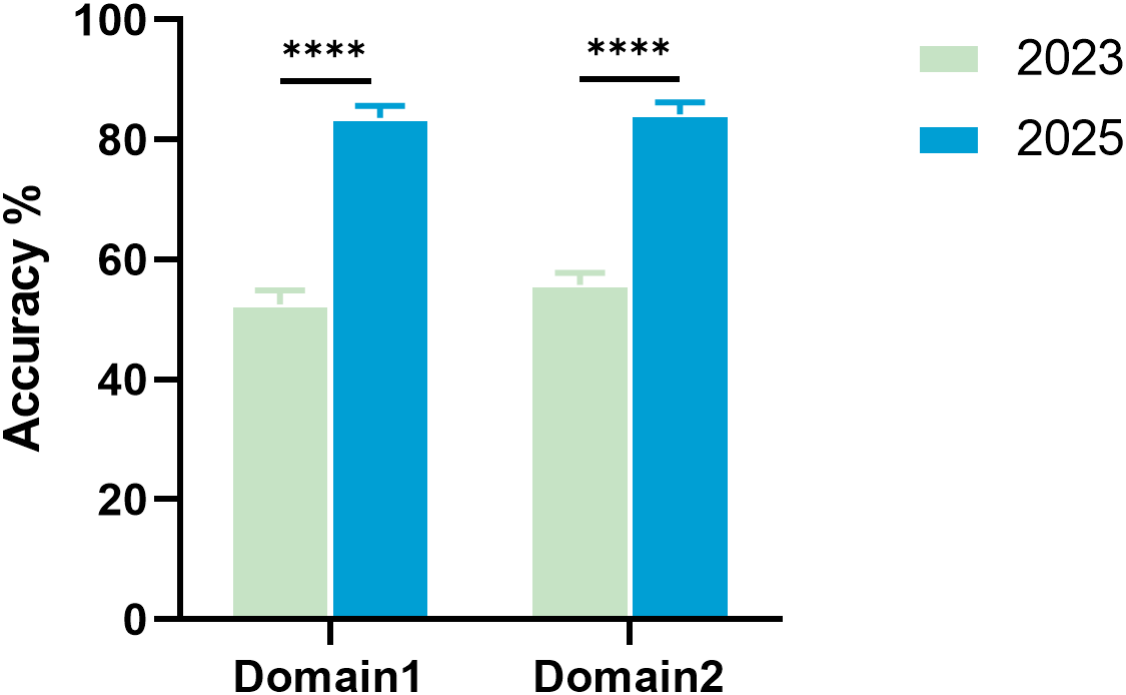
Improvement in diabetes management knowledge among team members following the implementation of management. Domain 1: diabetes treatment and complication management; Domain 2: comprehensive control goals and lifestyle intervention. The mean accuracy (%) of the management team members in assessments encompassing various modules of diabetes management knowledge were evaluated during the preliminary training phase prior to the commencement of the study in 2023 and at the study’s conclusion in 2025. ****P<0.0001.

In this study, comprehensive assessments were conducted to evaluate the knowledge proficiency of diabetes management team members both prior to and following the implementation of the management intervention. The evaluation encompassed two domains: “Diabetes Treatment and Complication Management (Domain 1)” and “Comprehensive Control Goals and Lifestyle Intervention (Domain 2)”. The results revealed a significant increase in the pass rate from 37.0% to 85.7%. The accuracy in Domain 1 improved from 52.56% to 83.60%, and that in Domain 2 improved from 55.79% (all P < 0.001; **Figure 5**).

## Discussion

Diabetes management in China, especially in rural and economically disadvantaged western regions, has historically encountered numerous challenges. These challenges include limited access to medical resources, inadequate primary care service capacity, generally low patient education levels, and considerable obstacles in promoting lifestyle modifications. The results of this study demonstrate that implementation of the telehealth management model in three townships of Dafang County, a resource-limited rural area in Guizhou Province, led to significant improvements in glucose metabolism indicators among local patients with diabetes and a reduction in the incidence of new chronic complications. Furthermore, the implementation of this telehealth management model significantly enhanced the knowledge and proficiency of township medical staff in diabetes treatment, control, and lifestyle interventions.

Previous studies have shown that integrated management effectively improves glycaemic control in diabetes patients. In Shanghai, the hospital-community cascade model significantly increased glycaemic control rates and complication screening coverage (16). Similarly, in Yiwu City, Zhejiang Province, the county-level integrated medical communities model achieved diabetes management and glycaemic control rates of 86.96% and 54.01%, respectively, outperforming other areas in the province (17).

The findings of this study align with the previously discussed evidence and offer more comprehensive metabolic indicators. Participants in the management group exhibited pronounced reductions in fasting blood glucose, 2-hour postprandial blood glucose, glycated haemoglobin, and insulin resistance levels. The overall lipid profiles improved, as evidenced by statistically significant decreases in TG levels and increases in HDL-C levels. Additionally, there were significant reductions in both blood pressure and BMI among the patients. Although the mean levels of glucose metabolism indicators in the management group at the conclusion of the study did not reach the target control levels (<7.0 mmol), they demonstrated substantial improvement relative to both the control group and their own baseline measurements. In this study, the initial proportion of chronic complications was greater in the management group than in the control group (65.9% vs. 46.5%), potentially attributable to regional disparities in foundational health services. After adjustment for variables in the multivariable analysis and PSM-DID analysis, the management group remained an independent factor associated with improved metabolic indicators and a reduction in complications.

In investigations of diabetes management models, developed countries often use a chronic disease framework centred on family physicians. In the U.S., primary care teams provide ongoing advice on medication, lifestyle, exercise, and diet (18). In developing countries where medical resources are unevenly distributed, such as China and India, diabetes management increasingly relies on remote technological methods to facilitate patient education and guidance while promoting lifestyle interventions (19). In the resource-abundant eastern regions of China, investigations into integrated diabetes management models have demonstrated promising results. For instance, a pilot study conducted by Peng et al. (20) within the Jiangsu Binhai county-level integrated medical communities model revealed that integrated health management interventions substantially increased self-management behaviours (SMB) and quality of life (QoL) scores among diabetes patients. Additionally, the “specialist-general practitioner-health manager” team-based nursing model implemented in Xiamen City significantly increased the diabetes consultation rate at community health service centres and facilitated regular follow-up and management of patients at the primary care level (21).

In this study, which was conducted in Dafang County, Guizhou Province, a telehealth management model was systematically developed. A key to its implementation was the establishment of an online collaborative working mechanism via a telehealth service platform, supported by a set of standardized specifications for personnel and equipment designed to work in conjunction with the platform. The model has several critical features that enhance its implementation and efficacy. First, it empowers county-level experts to deliver online instruction and guidance, thereby continuously and directly improving the professional competencies of rural health care providers. Second, it facilitates online collaborative diagnosis and treatment among medical personnel across all three tiers, enabling them to jointly develop and refine patient management plans while providing self-management support. Third, the model is designed for practical scalability. Its hardware requirements are largely adaptable, with only the core video conferencing network being essential. Each primary care institution requires only one staff member for platform management, thus placing minimal demands on human and material resources. This low-threshold design renders the model particularly suitable for promotion in resource-limited settings, especially in regions where health care workers face constraints in knowledge and skills related to the treatment and management of chronic diseases such as diabetes, thereby effectively addressing this specific gap.

The design of this study was predominantly informed by “National Guidelines for the Prevention and Control of Diabetes in Primary Care (2022)” (22). A comparison between the 2022 guidelines and the newly released 2025 update (23) reveals that the latter incorporates additional content on patient self-management support within the “Diabetes Health Management” section and imposes more stringent requirements for primary health care institutions to deliver systematic health education to patients. The telehealth management model developed in this study demonstrates substantial alignment with the updated guidelines and represents an innovative practical application in rural western China. Initial implementation suggests that this model significantly enhances the overall effectiveness of diabetes management in these regions and offers a viable framework for adhering to the guidelines’ requirements regarding self-management support and health education.

This study is subject to several limitations. As an exploratory quasi-experimental investigation, it is constrained by a limited sample size and the absence of complete randomization in group allocation, which may lead to residual confounding despite the implementation of methods such as propensity score matching combined with difference-in-differences (PSM-DID) to mitigate biases. Additionally, the study does not include an assessment of patients’ self-management behaviours, quality of life, or psychological status.

In conclusion, the telehealth management model developed in this study and implemented in Dafang County, Guizhou Province, effectively improved patients’ metabolic indicators, reduced their risk of complications, and enhanced primary health care service capacity. These findings offer insight for future large-scale randomized controlled trials and provide a replicable example for the scalable implementation of integrated diabetes management in similar underdeveloped regions.

## Data Availability

The original contributions presented in the study are included in the article/**Supplementary Material**. Further inquiries can be directed to the corresponding authors.

## Supplementary material

Supplementary Data Details of the telehealth management model.

Supplementary Table S1 Results of the Multivariable Linear Regression Analysis for Endpoint FBG.

Supplementary Table S2 Results of the Multivariable Linear Regression Analysis for Endpoint 2hPG.

Supplementary Table S3 Results of the Multivariable Linear Regression Analysis for Endpoint HbA1c.

Supplementary Table S4 Results of the Multivariable Logistic Regression Analysis for New-onset Chronic Complications.

Supplementary Table S5 The SMD and VR of the Three Matching Methods.

Supplementary Table S6 Raw Data for the DID Analysis (Optimal Full Matching).

Supplementary Table S7 Raw Data for the DID Analysis (1:4 Nearest Neighbour Matching).

Supplementary Table S8 Raw Data for the DID Analysis (Optimal Pair Matching).

Supplementary Table S9 Results of the Parallel Trends Assumption Test for FBG.

Supplementary Table S10 Results of the Parallel Trends Assumption Test for 2hPG.

Supplementary Table S11 Results of the Parallel Trends Assumption Test for HbA1c.

Supplementary Figure S1 Subgroup analysis of the association between 2hPG postintervention and diabetes management.

Supplementary Figure S2 Subgroup analysis of the association between HbA1c postintervention and diabetes management.

Supplementary Figure S3 Histogram and density plots of the propensity scores (1:4 nearest neighbour matching) for the control and management groups.

Supplementary Figure S4 Histogram and density plots of the propensity scores (optimal pair matching) for the control and management groups.
