# Supplementary material for: Evaluating the efficacy of a telehealth management model for chronic diabetes in resource-constrained regions
Source: Front Endocrinol (Lausanne). 2026 Apr 23;17:1812377. doi: 10.3389/fendo.2026.1812377 (PMC13149080; doi:10.3389/fendo.2026.1812377)
Supplement: Supplementary Data — Details of the telehealth management model. [file DataSheet1.zip › Supplementary Tables.docx]

Table S1: Results of Multivariable Linear Regression Analysis for Endpoint FBG

| **Variable** | *b* value (95%CI) | *β* | *t* | *P* value |
| --- | --- | --- | --- | --- |
| Diabetes management | -1.91(-2.98,-0.84) | -0.219 | -3.527 | 0.001 |
| Sex | -0.31(-1.85,1.23) | -0.036 | -0.396 | 0.692 |
| Age | -0.02(-0.08,0.04) | -0.039 | -0.591 | 0.556 |
| Ethnicity | -1.31(-2.73,0.11) | -0.115 | -1.827 | 0.069 |
| Educational attainment | 0.01(-0.56,0.57) | 0.002 | 0.028 | 0.978 |
| Smoke | 0.35(-1.19,1.90) | 0.039 | 0.449 | 0.654 |
| FBG pre | 0.27(0.15,0.39) | 0.33 | 4.531 | <0.001 |
| 2hPG pre | 0.04(-0.07,0.15) | 0.055 | 0.68 | 0.497 |
| HbA1c pre | 0.04(-0.22,0.31) | 0.025 | 0.306 | 0.76 |
| Fasting insulin pre | 0.00(-0.01,0.01) | 0.036 | 0.572 | 0.568 |
| C-peptied pre | -0.30(-0.67,0.07) | -0.098 | -1.61 | 0.109 |
| TG pre | -0.06(-0.20,0.07) | -0.068 | -0.93 | 0.353 |
| TC pre | 0.24(-0.07,0.55) | 0.108 | 1.509 | 0.133 |
| LDL-C pre | 0.07(-0.07,0.21) | 0.057 | 0.932 | 0.352 |
| HDL-C pre | -0.46(-2.03,1.12) | -0.036 | -0.569 | 0.57 |
| BMI pre | -0.12(-0.26,0.02) | -0.114 | -1.662 | 0.098 |
| SBP pre | 0.03(-0.02,0.07) | 0.11 | 1.18 | 0.239 |
| DBP pre | -0.05(-0.12,0.03) | -0.116 | -1.183 | 0.238 |
| Baseline chronic complication | 0.53(-0.68,1.74) | 0.061 | 0.862 | 0.39 |
| Adherence to medical advice | 1.35(0.67,2.03) | 0.235 | 3.9 | 0.001 |

Table S2: Results of Multivariable Linear Regression Analysis for Endpoint 2hPG

| **Variable** | *b* value (95%CI) | *β* | *t* | *P* value |
| --- | --- | --- | --- | --- |
| Diabetes management | -3.41(-4.90,-1.93) | -0.259 | -4.546 | <0.001 |
| Sex | 0.89(-1.24,3.02) | 0.069 | 0.824 | 0.411 |
| Age | 0.03(-0.06,0.11) | 0.038 | 0.616 | 0.539 |
| Ethnicity | -1.79(-3.75,0.18) | -0.104 | -1.797 | 0.074 |
| Educational attainment | -0.72(-1.50,0.06) | -0.111 | -1.818 | 0.071 |
| Smoke | 1.09(-1.05,3.22) | 0.08 | 1.001 | 0.318 |
| FBG pre | 0.23(0.07,0.39) | 0.186 | 2.779 | 0.006 |
| 2hPG pre | 0.38(0.23,0.54) | 0.371 | 4.978 | <0.001 |
| HbA1c pre | -0.45(-0.82,-0.09) | -0.181 | -2.444 | 0.015 |
| Fasting insulin pre | 0.00(-0.02,0.01) | -0.039 | -0.665 | 0.507 |
| C-peptied pre | -0.01(-0.52,0.50) | -0.001 | -0.025 | 0.98 |
| TG pre | -0.05(-0.24,0.13) | -0.036 | -0.541 | 0.589 |
| TC pre | 0.25(-0.18,0.68) | 0.077 | 1.161 | 0.247 |
| LDL-C pre | 0.03(-0.17,0.22) | 0.016 | 0.279 | 0.78 |
| HDL-C pre | -1.88(-4.06,0.30) | -0.099 | -1.698 | 0.091 |
| BMI pre | -0.14(-0.33,0.06) | -0.086 | -1.37 | 0.172 |
| SBP pre | 0.01(-0.05,0.07) | 0.039 | 0.456 | 0.649 |
| DBP pre | -0.01(-0.11,0.09) | -0.017 | -0.191 | 0.849 |
| Baseline chronic complication | 1.23(-0.44,2.91) | 0.095 | 1.451 | 0.148 |
| Adherence to medical advice | 2.15(1.21,3.10) | 0.249 | 4.496 | <0.001 |

Table S3: Results of Multivariable Linear Regression Analysis for Endpoint HbA1c

| **Variable** | *b* value (95%CI) | *β* | *t* | *P* value |
| --- | --- | --- | --- | --- |
| Diabetes management | -1.55(-2.08,-1.01) | -0.309 | -5.647 | <0.001 |
| Sex | 0.81(0.03,1.59) | 0.165 | 2.06 | 0.041 |
| Age | 0.01(-0.02,0.04) | 0.034 | 0.576 | 0.565 |
| Ethnicity | -0.19(-0.90,0.53) | -0.029 | -0.524 | 0.601 |
| Educational attainment | -0.32(-0.61,-0.04) | -0.131 | -2.245 | 0.026 |
| Smoke | -0.90(-1.68,-0.12) | -0.175 | -2.277 | 0.024 |
| FBG pre | 0.13(0.07,0.19) | 0.271 | 4.226 | <0.001 |
| 2hPG pre | 0.06(0.00,0.11) | 0.147 | 2.063 | 0.04 |
| HbA1c pre | 0.08(-0.06,0.21) | 0.079 | 1.112 | 0.268 |
| Fasting insulin pre | 0.00(-0.01,0.00) | -0.057 | -1.02 | 0.309 |
| C-peptied pre | -0.21(-0.39,-0.02) | -0.117 | -2.179 | 0.031 |
| TG pre | -0.04(-0.11,0.03) | -0.072 | -1.12 | 0.264 |
| TC pre | 0.06(-0.10,0.21) | 0.045 | 0.715 | 0.476 |
| LDL-C pre | 0.01(-0.07,0.08) | 0.009 | 0.162 | 0.872 |
| HDL-C pre | -0.70(-1.49,0.10) | -0.096 | -1.727 | 0.086 |
| BMI pre | -0.05(-0.12,0.02) | -0.087 | -1.444 | 0.15 |
| SBP pre | -0.01(-0.03,0.01) | -0.061 | -0.738 | 0.461 |
| DBP pre | 0.00(-0.04,0.04) | -0.012 | -0.139 | 0.889 |
| Baseline chronic complication | -0.08(-0.69,0.53) | -0.017 | -0.27 | 0.788 |
| Adherence to medical advice | 1.03(0.69,1.38) | 0.315 | 5.928 | <0.001 |

Table S4: Results of Multivariable Logistic Regression Analysis for New-onset Chronic Complications

| **Variable** | *OR* (95%CI) | *B* | *SE* | *P* value |
| --- | --- | --- | --- | --- |
| Diabetes management | 0.25(0.09,0.69) | -1.384 | 0.518 | 0.008 |
| Sex | 0.93(0.25,3.49) | -0.072 | 0.674 | 0.915 |
| Age | 1.05(1.00,1.10) | 0.046 | 0.025 | 0.067 |
| Ethnicity | 1.24(0.41,3.76) | 0.219 | 0.564 | 0.699 |
| Educational attainment | 0.88(0.53,1.48) | -0.124 | 0.265 | 0.64 |
| Smoke | 2.13(0.56,8.10) | 0.757 | 0.681 | 0.266 |
| FBG pre | 0.99(0.90,1.09) | -0.011 | 0.05 | 0.832 |
| 2hPG pre | 1.05(0.97,1.14) | 0.049 | 0.043 | 0.26 |
| HbA1c pre | 1.01(0.80,1.26) | 0.005 | 0.115 | 0.964 |
| Fasting insulin pre | 1.00(0.99,1.01) | -0.001 | 0.004 | 0.869 |
| C-peptied pre | 1.08(0.85,1.38) | 0.081 | 0.123 | 0.512 |
| TG pre | 0.99(0.89,1.10) | -0.012 | 0.055 | 0.825 |
| TC pre | 1.00(0.75,1.32) | -0.004 | 0.144 | 0.978 |
| LDL-C pre | 0.95(0.78,1.16) | -0.052 | 0.101 | 0.61 |
| HDL-C pre | 0.81(0.21,3.10) | -0.21 | 0.685 | 0.759 |
| BMI pre | 0.96(0.85,1.08) | -0.043 | 0.063 | 0.488 |
| SBP pre | 1.04(1.00,1.08) | 0.04 | 0.018 | 0.029 |
| DBP pre | 0.94(0.88,1.00) | -0.061 | 0.033 | 0.064 |
| Baseline chronic complication | 0.15(0.05,0.42) | -1.934 | 0.538 | <0.001 |
| Adherence to medical advice | 2.27(1.33,3.87) | 0.818 | 0.273 | 0.003 |

Table S5: The SMD and VR of Three Matching Methods

|  | 1:4 Nearest neighbor matching | | Optimal pair matching | | Optimal full matching | |
| --- | --- | --- | --- | --- | --- | --- |
|  | SMD | VR | SMD | VR | SMD | VR |
| Age | -0.03 | 1.1012 | -0.0562 | 1.0218 | 0.208 | 0.8475 |
| Sex_female | -0.057 | \ | -0.057 | \ | 0.0144 | \ |
| Sex_male | 0.057 | \ | 0.057 | \ | -0.0144 | \ |
| Han_ethnicite | 0.0151 | \ | 0.0529 | \ | -0.0863 | \ |
| Ethnic_minority | -0.0151 | \ | -0.0529 | \ | 0.0863 | \ |
| Primary_school_and_below | -0.3889 | \ | -0.2211 | \ | 0.0498 | \ |
| Junior_middle_school | 0.1381 | \ | 0.0806 | \ | -0.1154 | \ |
| Senior_middle_school_and_above | 0.3254 | \ | 0.1830 | \ | 0.0542 | \ |
| Smoke_no | 0.1109 | \ | 0.0287 | \ | 0.0967 | \ |
| Smoke_yes | -0.1109 | \ | -0.0287 | \ | -0.0967 | \ |
| FBG_pre | 0.0234 | 0.8967 | 0.1163 | 1.0416 | 0.0690 | 1.1194 |
| 2hPG_pre | 0.2603 | 0.7339 | 0.1791 | 0.6901 | 0.0215 | 0.7924 |
| HbA1c_pre | 0.2334 | 0.8925 | 0.1701 | 0.8431 | -0.0958 | 0.775 |
| Fasting insulin_pre | 0.0752 | 1.068 | 0.0768 | 1.0228 | -0.2778 | 0.4296 |
| C_peptied_pre | -0.6092 | 0.0958 | -0.2386 | 0.1779 | -0.1237 | 0.264 |
| TG_pre | 0.0087 | 2.1706 | 0.0004 | 1.5122 | -0.0973 | 0.8677 |
| TC_pre | 0.0355 | 1.9959 | 0.0255 | 1.8013 | -0.0330 | 1.2705 |
| LDL_C_pre | 0.0776 | 0.0588 | 0.1474 | 0.1106 | 0.1823 | 0.1327 |
| HDL_C_pre | 0.0239 | 0.6965 | 0.0357 | 0.8299 | 0.0655 | 0.8568 |
| BMI_pre | 0.0152 | 0.7097 | -0.0233 | 0.6447 | 0.0142 | 0.5329 |
| SBP_pre | 0.1664 | 1.0755 | 0.0458 | 0.9900 | 0.0476 | 1.2367 |
| Baseline_complication_no | -0.4914 | \ | -0.2597 | \ | 0.0585 | \ |
| Baseline_complication_yes | 0.4914 | \ | 0.2597 | \ | -0.0585 | \ |
| Adherence_good | 0.3151 | \ | 0.1147 | \ | 0.0732 | \ |
| Adherence_fair | -0.2266 | \ | -0.0978 | \ | 0.0116 | \ |
| Adherence_poor | -0.1601 | \ | -0.0400 | \ | -0.1143 | \ |

Table S9: Results of Parallel Trends Assumption Test for FBG

| Variable | Estimate | Std. Error | t value | *P* value |
| --- | --- | --- | --- | --- |
| (Intercept) | 7.533202205 | 7.759300982 | 0.970860935 | 0.332942369 |
| group1 | -10.62544699 | 12.64716752 | -0.840144402 | 0.401960843 |
| age | -0.032540419 | 0.064913409 | -0.501289642 | 0.616790174 |
| sex1 | -1.287614239 | 1.807204894 | -0.712489349 | 0.477099966 |
| ethnic1 | -1.290979713 | 1.226801421 | -1.052313513 | 0.294090221 |
| educational_attainment2 | -1.00072383 | 1.786899346 | -0.560033687 | 0.576164548 |
| educational_attainment3 | -0.405959843 | 1.315022074 | -0.308709527 | 0.757905544 |
| smoke1 | 0.218086316 | 1.93287017 | 0.112830298 | 0.910292962 |
| 2hPG | 0.243272396 | 0.125255105 | 1.942215422 | 0.05369821 |
| HbA1c | 0.502213287 | 0.280087897 | 1.793056012 | 0.07467144 |
| Fasting insulin | -0.019328322 | 0.007517422 | -2.571137119 | 0.010958515 |
| C_peptide | 0.606995121 | 0.419850935 | 1.445739596 | 0.15001854 |
| TG | 0.011635034 | 0.097436122 | 0.119411913 | 0.905084447 |
| TC | 0.104033269 | 0.308235338 | 0.337512467 | 0.736130537 |
| LDL_C | 0.041866083 | 0.05773664 | 0.725121564 | 0.469335003 |
| HDL_C | -1.702409227 | 1.750924034 | -0.972291883 | 0.3322322 |
| BMI | -0.105419666 | 0.157539047 | -0.669165315 | 0.50426191 |
| SBP | -0.011512392 | 0.049570125 | -0.232244553 | 0.816616383 |
| DBP | 0.064558714 | 0.087799003 | 0.735301222 | 0.463129242 |
| Complication1 | -1.581105452 | 1.012349101 | -1.561818399 | 0.120116947 |
| group1:age | -0.086511549 | 0.089517199 | -0.966423774 | 0.335150779 |
| group1:sex1 | 3.81610022 | 2.437856561 | 1.565350596 | 0.119286818 |
| group1:ethnic1 | 1.314962834 | 1.672237676 | 0.786349245 | 0.432714414 |
| group1:educational_attainment2 | 0.038741323 | 2.343234504 | 0.016533268 | 0.986827583 |
| group1:educational_attainment3 | -0.417410906 | 1.847278004 | -0.225959983 | 0.821493254 |
| group1:smoke1 | -0.756312236 | 2.496062658 | -0.303002103 | 0.76224408 |
| group1:2hPG | 0.128691098 | 0.165880331 | 0.775806854 | 0.438898389 |
| group1:HbA1c | -0.301918135 | 0.408569656 | -0.738963676 | 0.460907857 |
| group1:Fasting insulin | 0.017767027 | 0.014581916 | 1.218428826 | 0.224682595 |
| group1:C_peptide | 1.778590222 | 1.478495398 | 1.20297312 | 0.23059336 |
| group1:TG | 0.048844576 | 0.187727457 | 0.260188767 | 0.795020594 |
| group1:TC | -0.225957826 | 0.394759703 | -0.572393342 | 0.567781843 |
| group1:LDL_C | 0.107344592 | 0.635499742 | 0.168913667 | 0.866057425 |
| group1:HDL_C | 3.812005009 | 3.173386898 | 1.201241806 | 0.23126235 |
| group1:BMI | 0.23404654 | 0.220760495 | 1.060183076 | 0.290505868 |
| group1:SBP | 0.047949038 | 0.059773766 | 0.80217529 | 0.42352727 |
| group1:DBP | -0.068820717 | 0.102867207 | -0.669024838 | 0.504351301 |
| group1:Complication1 | 2.747194832 | 1.433913834 | 1.915871628 | 0.056993148 |

Table S10: Results of Parallel Trends Assumption Test for 2hPG

| Variable | Estimate | Std. Error | t value | *P* value |
| --- | --- | --- | --- | --- |
| (Intercept) | 0.583912322 | 7.22655895 | 0.08080088 | 0.935691598 |
| group1 | 10.08864545 | 10.24950505 | 0.984305622 | 0.32630883 |
| age | 0.067402471 | 0.06832162 | 0.986546729 | 0.32521156 |
| sex1 | 3.992026549 | 1.732862831 | 2.303717569 | 0.022400673 |
| ethnic1 | -0.526268897 | 1.638219102 | -0.321244513 | 0.748404113 |
| educational_attainment2 | -1.225377388 | 1.523446321 | -0.804345628 | 0.422276408 |
| educational_attainment3 | -0.536492186 | 1.592865128 | -0.33680955 | 0.736659487 |
| smoke1 | 1.444733937 | 1.892277529 | 0.763489454 | 0.44618801 |
| FBG | 0.256346499 | 0.124842879 | 2.053352984 | 0.04150947 |
| HbA1c | 0.786604113 | 0.272203482 | 2.889765065 | 0.004337561 |
| Fasting insulin | -0.009656928 | 0.007076861 | -1.364577944 | 0.174118051 |
| C_peptide | -0.119327538 | 0.274971762 | -0.433962881 | 0.664843889 |
| TG | 0.013232763 | 0.105288422 | 0.125681083 | 0.900127017 |
| TC | -0.293533768 | 0.289688838 | -1.013272619 | 0.312313076 |
| LDL_C | 0.007970652 | 0.051825084 | 0.153799122 | 0.877943338 |
| HDL_C | -0.511034693 | 1.096679451 | -0.46598365 | 0.641800127 |
| BMI | 0.004264763 | 0.130964572 | 0.032564252 | 0.97405877 |
| SBP | -0.01596416 | 0.044161291 | -0.361496671 | 0.718159346 |
| DBP | 0.055559172 | 0.076967761 | 0.721849916 | 0.471339281 |
| Complication1 | 1.416227793 | 1.117109845 | 1.267760552 | 0.206548665 |
| group1:age | 0.026918378 | 0.090976217 | 0.295883679 | 0.76766576 |
| group1:sex1 | -6.865209126 | 2.225411197 | -3.084917131 | 0.002363129 |
| group1:ethnic1 | 0.477661049 | 2.349919529 | 0.203266981 | 0.839159851 |
| group1:educational_attainment2 | 2.132334554 | 2.202974304 | 0.967934374 | 0.334397875 |
| group1:educational_attainment3 | 1.697309337 | 2.00300858 | 0.847379963 | 0.397927712 |
| group1:smoke1 | -0.241952259 | 2.488790562 | -0.097216802 | 0.92266422 |
| group1:FBG | 0.055229488 | 0.158340005 | 0.348803122 | 0.727651683 |
| group1:HbA1c | 0.441186596 | 0.364668972 | 1.209827625 | 0.227958359 |
| group1:Fasting insulin | 0.016038408 | 0.010346785 | 1.550086106 | 0.122907018 |
| group1:C_peptide | -1.849513265 | 0.933718775 | -1.980803337 | 0.049162385 |
| group1:TG | -0.096002593 | 0.153474244 | -0.625529015 | 0.53242961 |
| group1:TC | 0.240775996 | 0.367523055 | 0.655131679 | 0.513233495 |
| group1:LDL_C | -0.084976554 | 0.479557943 | -0.177197678 | 0.859555728 |
| group1:HDL_C | -2.91582338 | 2.320577715 | -1.256507533 | 0.210587788 |
| group1:BMI | -0.184437062 | 0.186074025 | -0.991202624 | 0.322939733 |
| group1:SBP | -0.038228391 | 0.051678889 | -0.739729339 | 0.460444221 |
| group1:DBP | -0.009066833 | 0.089213723 | -0.10163047 | 0.919165031 |
| group1:Complication1 | -1.860765997 | 1.510479909 | -1.231903838 | 0.21961907 |

Table S11: Results of Parallel Trends Assumption Test for HbA1c

| Variable | Estimate | Std. Error | t value | *P* value |
| --- | --- | --- | --- | --- |
| (Intercept) | 9.908828403 | 3.335442494 | 2.970768772 | 0.003383303 |
| group1 | -5.543579004 | 4.445917726 | -1.246891946 | 0.214084588 |
| age | -0.051030584 | 0.022916101 | -2.226844113 | 0.027218807 |
| sex1 | 0.086271365 | 0.620873004 | 0.138951709 | 0.889646278 |
| ethnic1 | -0.646259026 | 0.619368635 | -1.04341581 | 0.298178742 |
| educational_attainment2 | -0.365968506 | 0.717750264 | -0.509882788 | 0.610768769 |
| educational_attainment3 | -0.828983347 | 0.503575372 | -1.646195172 | 0.101498034 |
| smoke1 | 0.307894437 | 0.695374008 | 0.442775302 | 0.658468905 |
| FBG | 0.157790468 | 0.051690408 | 3.052606365 | 0.002618498 |
| HbA1c | 0.106156675 | 0.05603738 | 1.894390386 | 0.059803952 |
| Fasting insulin | 0.011506721 | 0.002899932 | 3.967928524 | 0.000105206 |
| C_peptide | -0.424782351 | 0.257542919 | -1.649365291 | 0.100846436 |
| TG | 0.020086221 | 0.04147262 | 0.484324874 | 0.628754077 |
| TC | 0.223604943 | 0.126616821 | 1.765997131 | 0.07912031 |
| LDL_C | 0.003550185 | 0.019102082 | 0.185853281 | 0.852772638 |
| HDL_C | 0.097514439 | 0.647528813 | 0.15059475 | 0.880466885 |
| BMI | -0.028227467 | 0.05457581 | -0.517215713 | 0.605651318 |
| SBP | -0.017369661 | 0.019696638 | -0.881859142 | 0.379048977 |
| DBP | -0.012379753 | 0.035857626 | -0.345247437 | 0.7303183 |
| Complication1 | 1.339484017 | 0.539164369 | 2.484370431 | 0.013907422 |
| group1:age | 0.010131082 | 0.032806625 | 0.30881208 | 0.757827657 |
| group1:sex1 | 0.371713585 | 0.917504739 | 0.40513533 | 0.685867271 |
| group1:ethnic1 | 1.072267985 | 0.924957521 | 1.159261869 | 0.24791097 |
| group1:educational_attainment2 | 0.17452057 | 0.935888551 | 0.186475804 | 0.852285205 |
| group1:educational_attainment3 | 0.844983869 | 0.769527883 | 1.098054908 | 0.273671986 |
| group1:smoke1 | -0.096024457 | 0.983417792 | -0.097643604 | 0.922325781 |
| group1:FBG | 0.083552113 | 0.062632564 | 1.334004366 | 0.183916307 |
| group1:HbA1c | -0.073177202 | 0.074155452 | -0.986808117 | 0.32508374 |
| group1:Fasting insulin | -0.013549714 | 0.004472094 | -3.029836358 | 0.002813489 |
| group1:C_peptide | 0.049291799 | 0.412396846 | 0.119525159 | 0.904994863 |
| group1:TG | 0.021167076 | 0.064193729 | 0.32973744 | 0.741988262 |
| group1:TC | -0.296910807 | 0.156306003 | -1.899548327 | 0.059118672 |
| group1:LDL_C | 0.115006425 | 0.184115206 | 0.624643816 | 0.53300922 |
| group1:HDL_C | 0.992666144 | 1.164826018 | 0.852201212 | 0.395254032 |
| group1:BMI | -0.001307658 | 0.072660473 | -0.017996833 | 0.98566165 |
| group1:SBP | 0.036722521 | 0.0233083 | 1.575512638 | 0.116923829 |
| group1:DBP | 0.004737773 | 0.041687377 | 0.113650052 | 0.909644014 |
| group1:Complication1 | -0.393394538 | 0.659606352 | -0.596408049 | 0.551664667 |
