# Supplementary material for: Evaluating the efficacy of a telehealth management model for chronic diabetes in resource-constrained regions
Source: Front Endocrinol (Lausanne). 2026 Apr 23;17:1812377. doi: 10.3389/fendo.2026.1812377 (PMC13149080; doi:10.3389/fendo.2026.1812377)
Supplement: Supplementary Data — Details of the telehealth management model. [file DataSheet1.zip › Supplementary Data.docx]

Details of the integrated management model

**Appendix 1. Requirements for Establishing the Telemedicine Service Management Platform**

1. Personnel Requirements: A telemedicine center is to be established within the county-level medical institution, tasked with overseeing the daily operations and management of telemedicine services. This center must be staffed by 2-3 dedicated administrators. Additionally, each primary care institution, including township health center and community health service center is required to establish a telemedicine room, which must be staffed by at least one full-time or part-time administrator.

2. Site Requirements

2.1 Teleconsultation Room Specifications

2.1.1 County-Level Medical Institutions

(1) A dedicated consultation room with a floor area of no less than 15 square meters shall be provided.

(2) Room temperature shall be maintained between 22–26°C, with humidity below 80%. The ambient noise level shall be 40 dB(A).

(3) Soundproof doors with a sound insulation rating of >50 dB shall be installed at the entrance/exit. The indoor ambient noise level shall be 40 dB(A).

(4) Video conferencing network access equipment shall be centrally installed in the hospital server room. The consultation room must be equipped with a network interface for connecting to the video conferencing network.

(5) The video camera shall be mounted at the center of the wall in the consultation room, with a vertical height of 1.2 to 1.6 meters from the floor.

(6) The display screen area and the camera shall be located on the same vertical plane.

2.1.2 Primary Care Institutions

(1) A dedicated consultation room with a floor area of no less than 10 square meters shall be provided.

(2) Air conditioning equipment shall be installed to maintain room temperature between 22–26°C, with humidity below 80%.

(3) The consultation room shall have soundproofing measures in place.

(4) The consultation room must be equipped with a network interface for connecting to the video conferencing network.

(5) The video camera shall be mounted at the center of the wall in the consultation room, with a vertical height of 1.2 to 1.6 meters from the floor.

(6) The display screen area and the camera shall be located on the same vertical plane.

2.2 Requirements for Telediagnosis Sites

Telediagnosis sites must be equipped with network access ports connecting to the healthcare private network via the hospital's local area network (LAN). Video equipment and software shall be provided as needed.

2.3 Requirements for Teletraining Sites

Medical institutions shall provide a teletraining site of appropriate scale. Depending on the actual situation of the institution, an independent teletraining site may be established, or the teleconsultation room may be used jointly for this purpose.

3. Facility Requirements

3.1 Teleconsultation Center/Room Equipment

3.1.1 County-Level Medical Institutions

High-definition audio-video interactive terminal, high-definition televisions (2 units), network switch, router, professional medical display monitor, document camera (overhead scanner), physician workstation, monochrome laser printer/scanner/copier multifunction device.

3.1.2 Primary Care Institutions

High-definition audio-video interactive terminal, high-definition televisions (2 units), network switch, router, document camera (overhead scanner), physician workstation, monochrome laser printer/scanner/copier multifunction device.

3.2 Telediagnosis Equipment

County-level hospitals shall be equipped with the necessary devices for teleradiology, tele-electrocardiography, and remote laboratory quality control.

Primary care institutions shall, at a minimum, be equipped with digital medical devices including Digital Radiography (DR) system, dynamic electrocardiograph (Holter monitor), static electrocardiograph, color Doppler ultrasound system, fully automated biochemical analyzer, blood cell counter, urine analyzer, and electrolyte analyzer.

3.3 Teletraining Equipment

High-definition audio-video interactive terminal, high-definition display device, high-fidelity audio equipment, workstation, etc.

**Appendix 2. Teleconsultation Workflow**

**
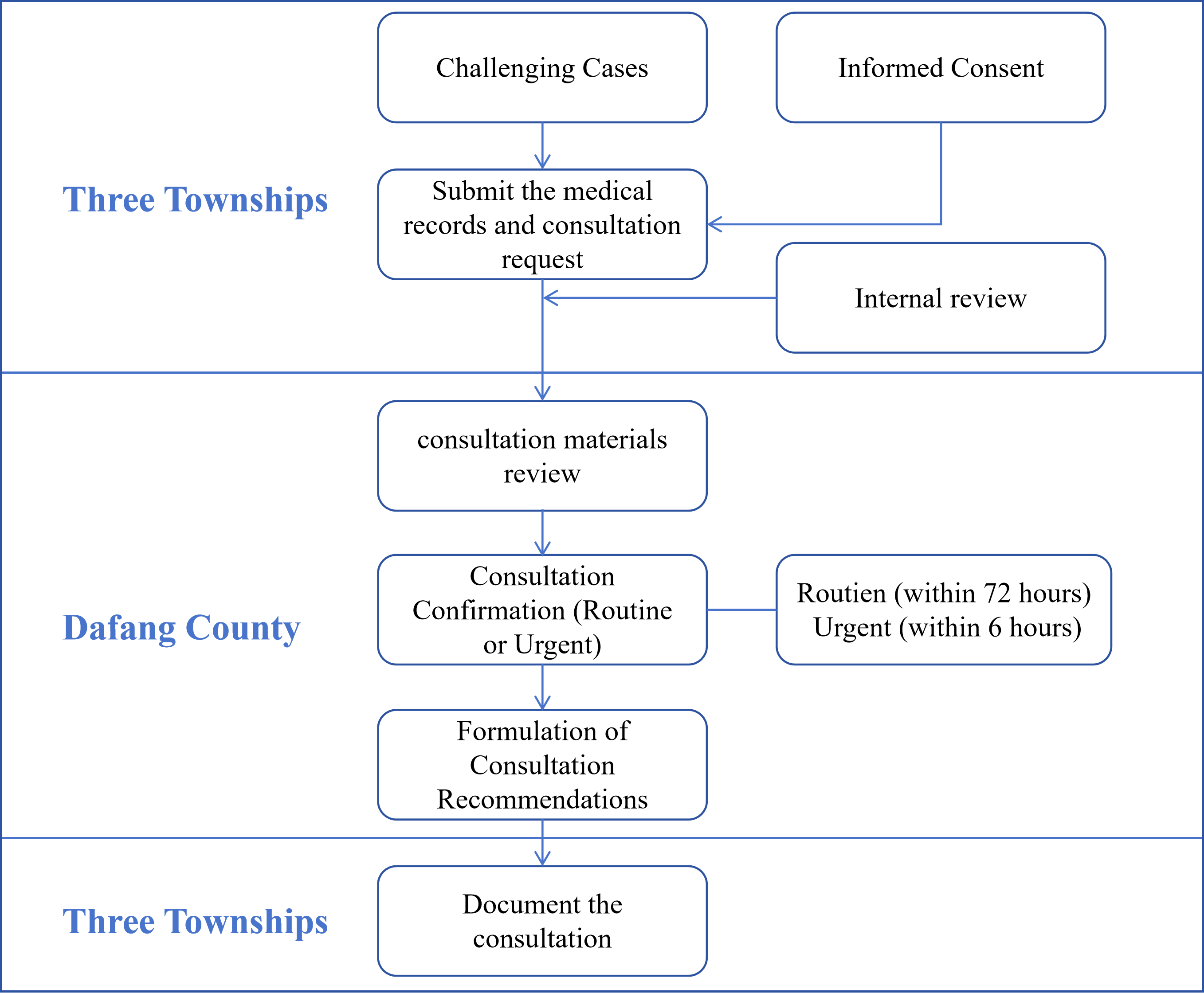
**

**Appendix 3. Teletraining Workflow**

**
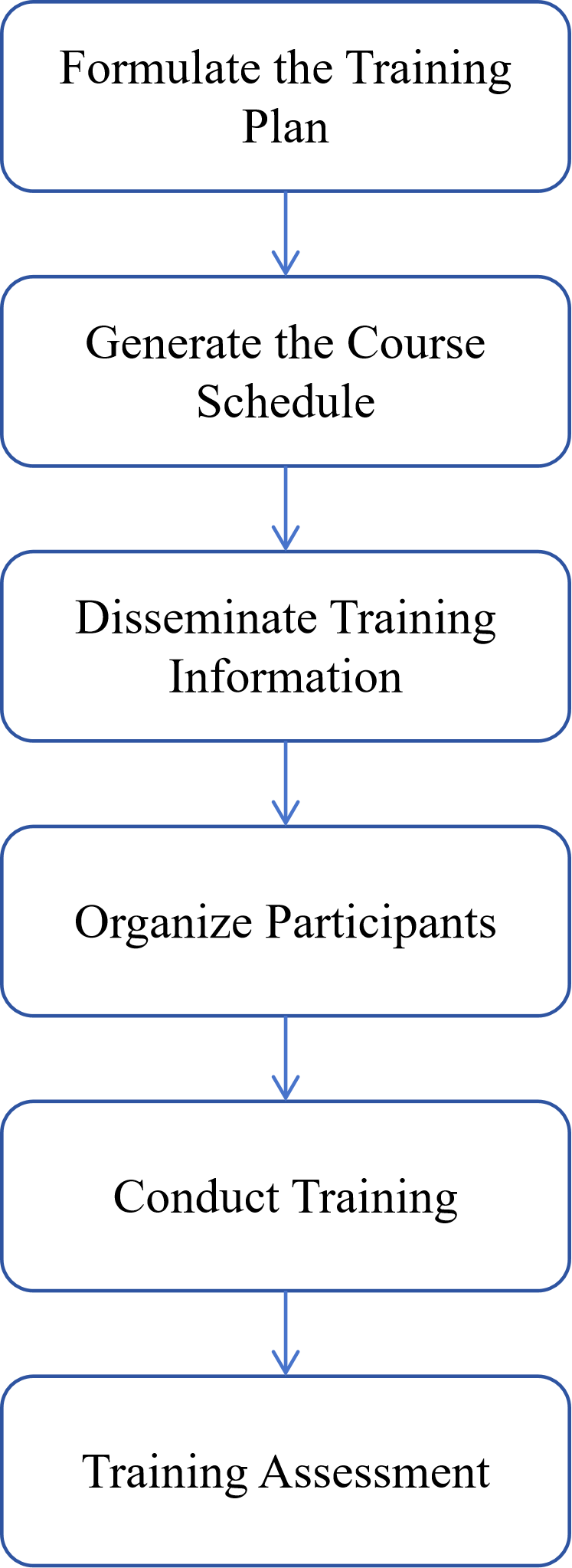
**
