# Supplementary figures and images for: Evaluating the efficacy of a telehealth management model for chronic diabetes in resource-constrained regions
Source: Front Endocrinol (Lausanne). 2026 Apr 23;17:1812377. doi: 10.3389/fendo.2026.1812377 (PMC13149080; doi:10.3389/fendo.2026.1812377)

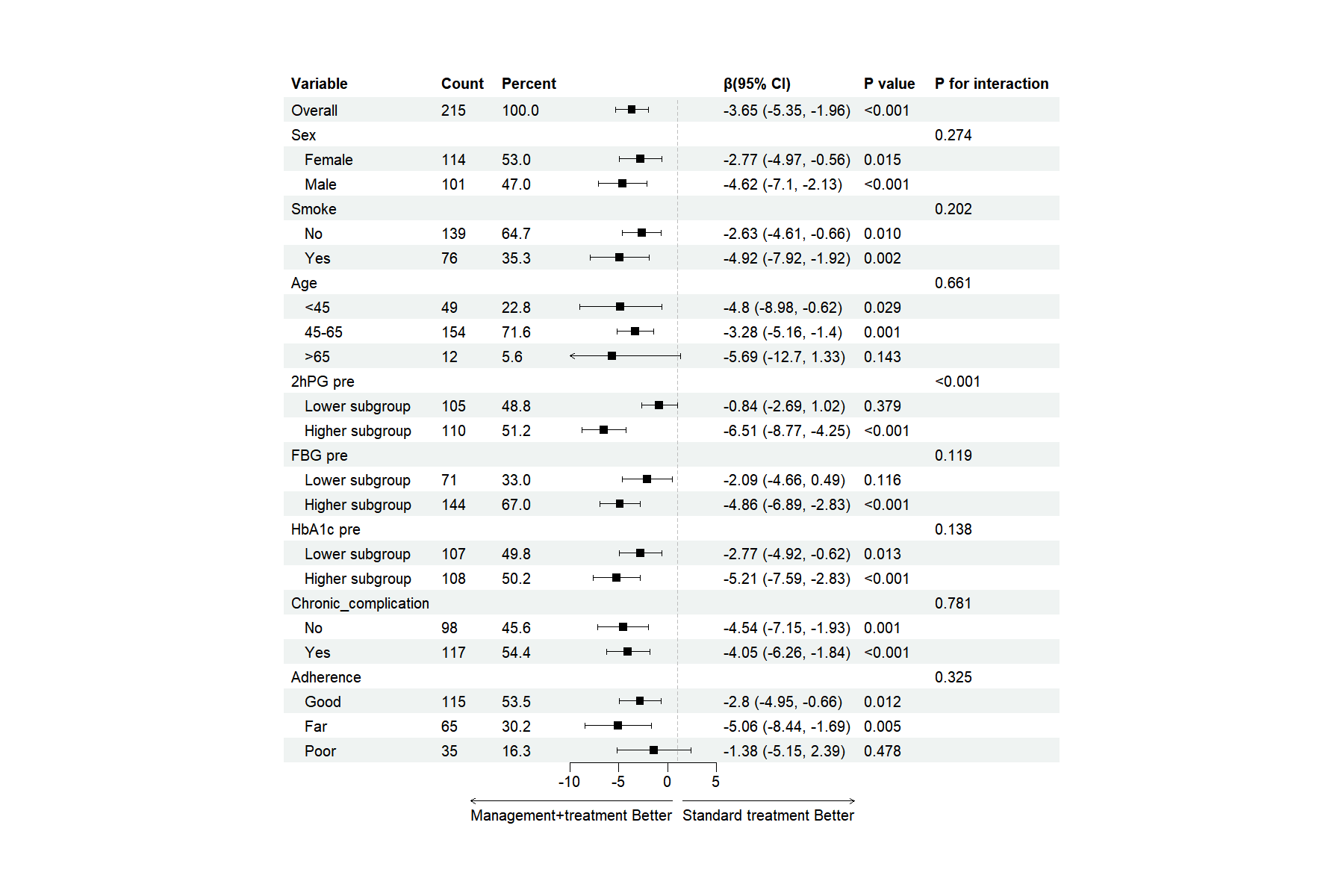

Supplement: Supplementary Data — Details of the telehealth management model. [file DataSheet1.zip › Figure S1.tiff]

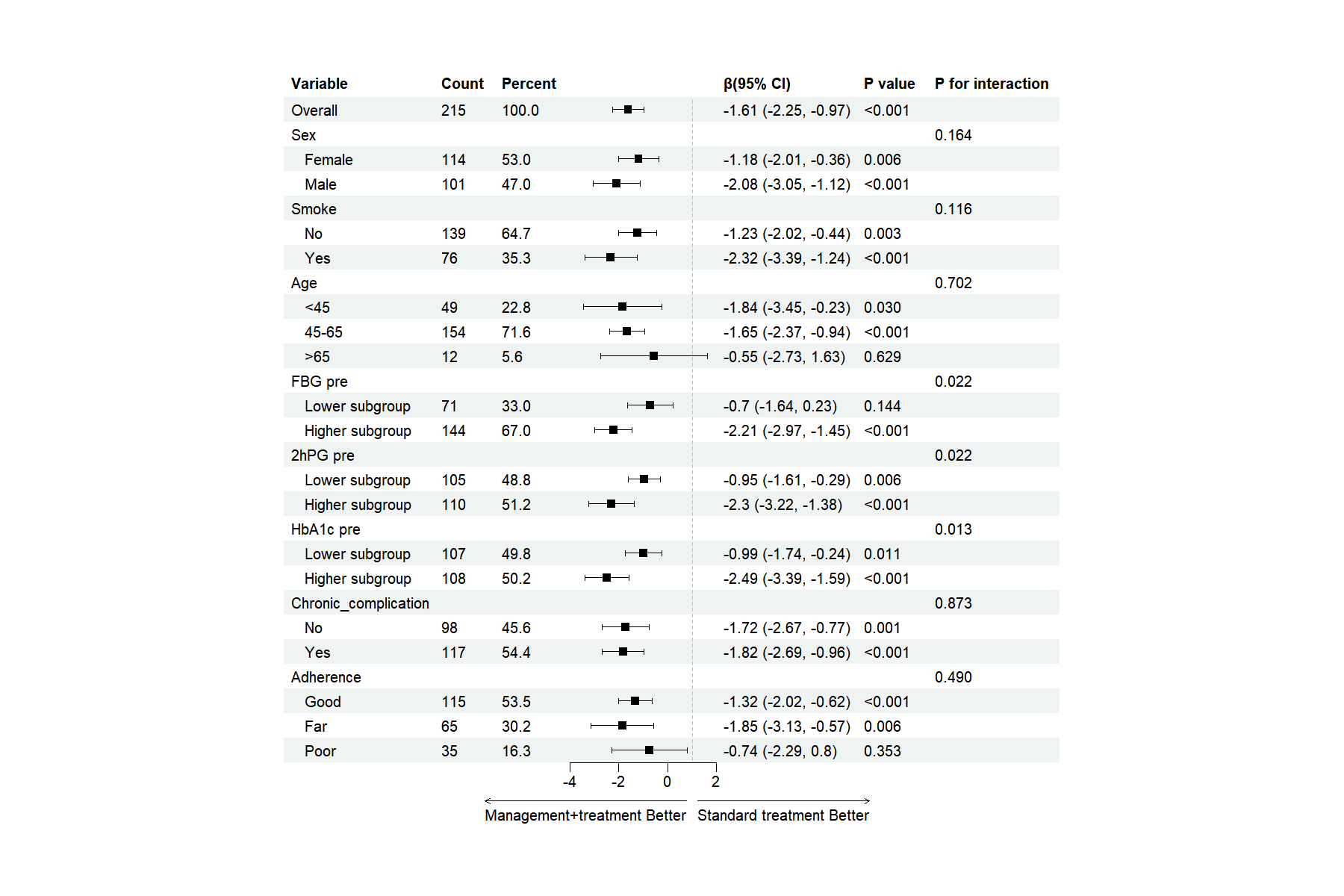

Supplement: Supplementary Data — Details of the telehealth management model. [file DataSheet1.zip › Figure S2.tiff]

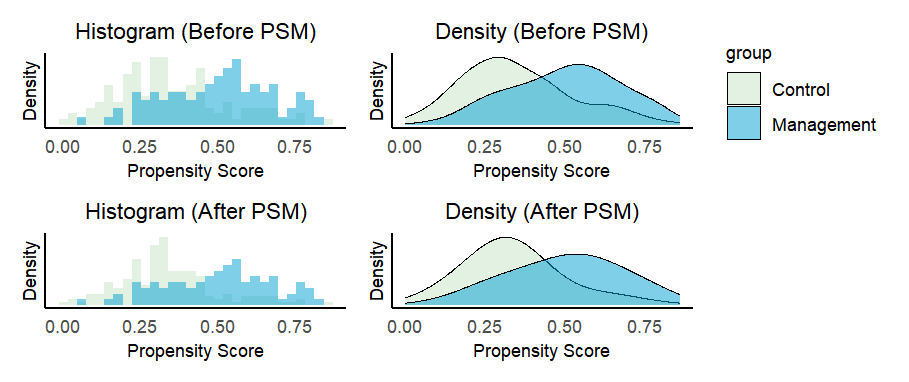

Supplement: Supplementary Data — Details of the telehealth management model. [file DataSheet1.zip › Figure S3.tiff]

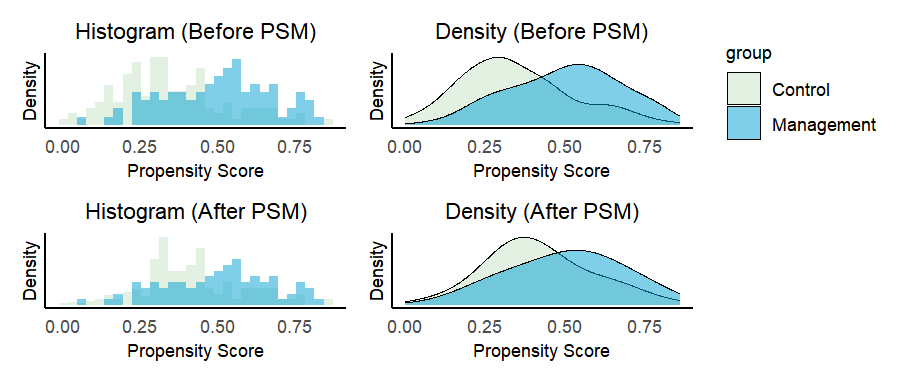

Supplement: Supplementary Data — Details of the telehealth management model. [file DataSheet1.zip › Figure S4.tiff]
